# Integrative analyses of 16S rDNA sequencing and serum metabolomics demonstrate significant roles for the oral microbiota and serum metabolites in post-kidney transplant diabetes mellitus

**DOI:** 10.1128/spectrum.00892-25

**Published:** 2025-06-10

**Authors:** Chao Liu, Sheng Chao, Lei Jia, Qizhen Yang, Qian Chen, Yulin Niu

**Affiliations:** 1 Department of Organ Transplantation, Affiliated Hospital of Guizhou Medical University 74720, Guiyang, Guizhou, China; 2 Urinary Surgery, The Affiliated Hospital of Guizhou Medical University 74720, Guiyang, Guizhou, China; Cleveland Clinic Lerner Research Institute, Cleveland, Ohio, USA

**Keywords:** post-transplant diabetes mellitus, oral microbiota, serum metabolites, kidney transplantation

## Abstract

Oral microbiota and serum metabolites play crucial roles in diabetes, but their relationship with post-transplant diabetes mellitus (PTDM), a common complication post-kidney transplantation, is not well characterized. This study investigated the relationship of oral microbiota and serum metabolites with PTDM using integrative analysis of 16S rDNA sequencing and serum metabolomics. We recruited 61 kidney transplant recipients, including 30 in the PTDM group and 31 in normal glucose tolerance controls. Oral samples and serum samples were collected from all the kidney transplant patients to perform 16S rDNA sequencing and serum metabolomics analysis. We annotated 689 oral microbial species, including 134 species unique to the PTDM group and 157 species unique to the control group. PTDM group showed upregulation of 36 metabolites and downregulation of 19 metabolites. Based on the random forest machine learning algorithm, genera such as *UCG−005* (AUC = 0.9355), *Succinivibrio* (AUC = 0.8108); *Akkermansia* (AUC = 0.7742), *Anaerovibrio* (AUC = 0.2667), and *Schwartzia* (AUC = 0.2667), and serum metabolites such as LPI 18:0 (AUC: 0.8086), methylglyoxal (AUC: 0.7946), Vulgarin (AUC: 0.7828), 2-mercaptobenzothiazole (AUC: 0.7591), and PI(18:0/20:3(5Z,8Z,11Z)) (AUC: 0.7419) showed high diagnostic potential and may serve as clinical biomarkers. Furthermore, clinical indicators in PTDM patients, such as creatinine, cystatin C, and urea, showed a significant association with the differential oral microbiota and serum metabolites. Dysbiosis in the oral microbiota of the PTDM patients was associated with changes in the serum metabolites and alterations in their functions. These findings provide new insights toward identifying mechanisms by which oral microbiota and serum metabolites contribute to the development of PTDM.

**IMPORTANCE:**

This study reveals an imbalance in oral microbiota in patients with post-transplant diabetes and uncovers the potential relationship between oral microbiota and serum metabolites. These findings provide new insights into the role of oral microbiota and serum metabolites in the treatment of post-transplant diabetes, offering relevant biomarkers for clinicians and future research.

## INTRODUCTION

Kidney transplantation is currently the best treatment option for patients with end-stage renal disease and significantly improves their quality of life compared to dialysis (1–3). However, patients undergoing kidney transplantation are at a significant risk of developing complications such as post-transplant diabetes mellitus (PTDM) (4–6). The incidence of PTDM ranges from 10% to 40% (7–9). PTDM affects patient survival and also increases the risk of complications such as acute rejection, infections, and cardiovascular diseases (10–12). PTDM is characterized by reduced insulin secretion and insulin resistance, which are also manifested in type 2 diabetes mellitus (T2DM) (13). The occurrence of PTDM is often closely related to factors such as obesity, dyslipidemia, a history of diabetes, immunosuppressive therapy, and hepatitis B virus infection (5, 14). However, the pathogenesis of PTDM remains unclear, thereby complicating its treatment and prevention.

Several studies have clearly established that the oral microbiota and serum metabolites significantly influence the occurrence and progression of glucose metabolism disorders (15, 16). For example, the relative abundance of *Prevotella* is positively associated with insulin resistance, whereas the relative abundance of Actinomyces spp. is negatively associated with insulin resistance (17). Previous studies have reported that reduced oral microbial diversity is potentially associated with insulin resistance and abnormal glucose metabolism (17, 18). A metabolomics study by Li et al. showed that the levels of cadaverine and L-(+)-leucine in the saliva were significantly higher in patients with T2DM than in the healthy subjects, and these elevated levels were also observed in the supragingival plaques of patients with T2DM compared to those in the healthy subjects (19). Oral microorganisms can also influence host metabolic processes by synthesizing and secreting specific metabolites (20). Recent studies have primarily focused on the link between diabetes, oral microbes, and their associated serum metabolites. However, the links between oral microbes, their associated serum metabolites, and PTDM have not been reported. Therefore, we hypothesized that the composition and functions of oral microbes and their associated serum metabolites are altered in patients with PTDM.

In recent years, significant advances in 16S rDNA sequencing, metagenomics, and metabolomics have assisted in unraveling the relationship between microbiomes and host diseases following kidney transplantation (21–23). Serum metabolomics involves the qualitative and quantitative analyses of all small molecular weight metabolites in the blood serum samples (24). This enables pathway analysis of differential metabolites to gain insights into the changes and patterns of endogenous metabolites in the organisms because of internal and external factors. On the other hand, 16S rDNA sequencing is a technique used to study the composition of microbial communities in samples and allows researchers to study the microbial diversity, richness, and structural relationships and investigate the interactions between microbes and hosts (25). Therefore, combining serum metabolomics with 16S rDNA sequencing technology provides a powerful approach to investigate the relationships between metabolites and microbes in the host organism (26, 27).

This study aimed to combine 16S rDNA sequencing with serum metabolomics to investigate alterations in the composition and diversity of oral microbiota and serum metabolites in the PTDM patients compared to the healthy individuals. Machine learning was used to identify biomarkers from the oral microbiota and serum metabolites and perform combinatorial analyses of differential metabolites and microbes with the clinical indicators. Understanding the relationships between oral microbiota, serum metabolites, and PTDM would provide new insights for the prevention and treatment of PTDM.

## MATERIALS AND METHODS

### Study subjects

This research study was approved by the Ethics Committee of the Affiliated Hospital of Guizhou Medical University (Approval Number: 2025158) and conducted in accordance with the Declaration of Helsinki guidelines. All the participating patients provided informed consent and signed consent forms. This study included 61 kidney transplant patients who underwent transplantation between October 2023 and October 2024, including 30 diagnosed with PTDM (PTDM group) and 31 with normal blood glucose levels after kidney transplantation (control group). The basic details and clinical indicators of the patients were obtained from the electronic medical record system of the Affiliated Hospital of Guizhou Medical University. The diagnostic criteria for PTDM were based on the relevant guidelines from ADA (American Diabetes Association) and WHO (World Health Organization) (28), which were as follows: (i) Fasting blood glucose >7 mmol/L; (ii) Random blood glucose >11.1 mmol/L with accompanying diabetes symptoms; and (iii) Blood glucose level ≥11.1 mmol/L 2 hours after a 75 g oral glucose tolerance test or HbA1C > 6.5%. The exclusion criteria were as follows: (i) Combined organ transplantation; (ii) Poor patient compliance or voluntary withdrawal from the study; (iii) Missing clinical data; (iv) Biopsy-confirmed acute rejection within the last 3 months; and (v) Graft loss or recipient death within 1 year post-transplant.

### Sample collection

All the subjects were required to fast and refrain from drinking, consuming alcohol, smoking, or chewing gum for at least 1 hour before sampling. Saliva samples were collected using a continuous collection process for one minute and were repeated multiple times until 2–5 mL of saliva (excluding any foamy portion) was obtained and placed in a sterile EP tube. The samples were placed in an insulated box designed for low temperatures, transported to the laboratory, and stored at −80°C. Blood samples were also collected under fasting conditions. The study subjects provided samples in the morning while fasting. Whole blood samples (4–6 mL) were collected in the vacuum blood collection tubes and sent to the laboratory. The blood was centrifuged at a speed of 1,800 × *g* for 10 minutes to separate the serum from the blood cells. The isolated serum was placed into a 1.5 mL centrifuge tube and spun at 13,000 × *g* for 2 minutes. Finally, the supernatant was transferred into a 1.5 mL cryogenic tube and stored at −80°C.

### 16S DNA sequencing

Total DNA was extracted from the oral microbiota using the cetyltrimethylammonium bromide (CTAB) method according to the manufacturer’s instructions and quantified using a Qubit fluorometer. Subsequently, the 16S rDNA gene was PCR amplified using universal primers 341F (5′-CCTACGGGNGGCWGCAG-3′) and 805R (5′-GACTACHVGGGTATCTAATCC-3′). The amplification protocol included initial denaturation at 98°C for 30 seconds, followed by 32 cycles of denaturation at 98°C for 10 seconds, annealing at 54°C for 30 seconds, and extension at 72°C for 45 seconds. The final extension step was performed at 72°C for 10 minutes. The PCR products were purified using AMPure XT Beads and re-quantified using the Qubit fluorometer to ensure that they met the requirements for library construction. Finally, the quality of the PCR products and the libraries was determined using an Agilent 2100 Bioanalyzer and the Illumina library quantification kit, and the sequencing was performed using the Illumina NovaSeq 6000 platform (29, 30).

The Cutadapt (v1.9) software was used to process the demultiplexed sequences by removing the sequencing primers. The paired-end reads were then merged using the FLASH (v1.2.8) software. The sliding window algorithm in fqtrim (v0.94) was used to trim the low-quality reads (quality score <20), short reads (length <100 bp), and records containing more than 5% “N” to ensure data quality. Quality filtering based on the fqtrim standards produced high-quality clean tags. Then, the Vsearch software (v2.3.4) was used to filter chimeric sequences, and the DADA2 package was used for denoising and generating the amplicon sequence variants (ASV). Furthermore, we used the SILVA and NT-16S databases and the QIIME2 plugin feature classifier for species annotation and sequence alignment. Finally, we performed alpha and beta diversity analyses using the QIIME2 software and calculated the relative abundance for bacterial classification. Details regarding the experimental reagents and instruments are listed in Tables S1 and S2.

### Serum metabolomics

We first subjected the collected serum samples to freeze-thaw treatment and extracted the metabolites using 80% methanol buffer. Briefly, 120 µL of pre-chilled 50% methanol was used to extract 20 µL of the sample. This was followed by vortexing for 1 minute and incubation at ambient temperature for 10 minutes. The extracted liquid was then stored at −20°C overnight. Subsequently, it was centrifuged at 4,000 × *g* for 20 minutes, and the supernatant was transferred to a 96-well plate. Prior to liquid chromatography-mass spectrometry (LC-MS) analysis, the samples were kept in a freezer at −80°C. Furthermore, 10 µL of samples from each extraction was pooled together to prepare a mixed quality control (QC) sample.

Chromatographic separation was conducted using the UltiMate 3000 UPLC system and an ACQUITY UPLC T3 column for reverse-phase analysis. The column was held at a temperature of 40°C, with the mobile phase consisting of 5 mM ammonium acetate and 5 mM acetic acid (solvent A) blended with acetonitrile (solvent B) at a flow rate of 0.3 mL/min. Metabolites were analyzed using a Q-Exactive high-resolution tandem mass spectrometer in both positive and negative ion modes. Precursor ion spectra were obtained at a resolution of 70,000 (for 70–1,050 m/z), with the automatic gain control (AGC) configured to 3e6 and a maximum injection time of 100 ms. In the data-dependent acquisition (DDA) mode, data collection was configured to prioritize the top three settings, and the fragment spectra were recorded at a resolution of 17,500 using an AGC of 1e5 and a maximum injection time of 80 ms. Furthermore, to ensure the stability and reliability of the entire collection process, a mixed quality control sample was collected after every 10 samples (31, 32).

The XCMS software was used for preprocessing the acquired mass spectrometry data, including peak picking, peak grouping, retention time correction, secondary peak grouping, and isotopic and adduct annotation (33). Initially, the LC-MS raw data files were transformed into the mzXML format. Subsequently, data processing was carried out using the XCMS, CAMERA, and metaX toolboxes in the R version 4.4.1 software (34). We characterized each ion by combining the retention time (RT) and m/z data and recording the intensity of each peak. This approach generated a three-dimensional matrix that included specific peak indices (retention time-m/z pairs), sample identifiers (observations), and ion intensity information (variables). Furthermore, we used the Kyoto Encyclopedia of Genes and Genomes (KEGG) and Human Metabolome Database (HMDB) online databases to annotate the metabolites and aligned the precise molecular mass data (m/z) from the samples with the corresponding information in these databases to verify the accuracy of our annotations. Details regarding the experimental reagents and instruments are listed in Tables S1 and S2.

### Integrated analysis of metabolomics and 16S rDNA sequencing

Based on the results of metabolomics and 16S rDNA sequencing, we screened for the differential metabolites and microbial taxa at the genus level using the following criteria: ratio (ratio) ≥1.2, *P*-value < 0.05, VIP >1, and *P* < 0.05. Subsequently, Spearman correlation analysis, Mantel tests, and Procrustes analysis were performed to determine the relationships between the differentially expressed metabolites and the differential microbial species. Finally, correlation heatmaps and network diagrams were generated to visualize the results. All the data analyses were performed using R version 4.4.1.

### Data processing and statistical analysis

Fisher’s exact test was used to determine the differentially abundant genera using *P* < 0.05 as the threshold parameter. The effect size was defined as linear discriminant analysis (LDA) ≥3.0 and *P* < 0.05, and Linear Discriminant Analysis Effect Size (LEfSe) analysis was performed using the nsegata-lefse software package. Other visualizations were generated using R (v3.4.4). Cluster heatmaps were created using the R pheatmap package. The principal component analysis (PCA) and significant differential protein analysis were performed using the R metaX package. Partial Least Squares Discriminant Analysis (PLSDA) was performed using the R ropls package, which also calculated the Variable Importance in Projection (VIP) scores. Correlation analysis was performed using the Pearson correlation coefficients, which were obtained from the R package “cor.” Metabolites were selected as significantly different if they satisfied all three following criteria: *P*-value < 0.05 from the *t*-test, fold change >1.2, and VIP scores from PLS-DA analysis. Differential enrichment analysis of the KEGG pathways was based on hypergeometric testing, where the functional entries with a statistical *P*-value < 0.05 were considered as significantly enriched for the differential proteins.

The Mann-Whitney U test (for non-normally distributed data) or the two-sample t-test (for normally distributed data) was used for comparing the continuous variables. Chi-square tests were used to compare the categorical variables. Normally distributed data were reported as mean ± standard deviation (SD), whereas skewed data were summarized as median and interquartile range (IQR). Categorical data were represented as percentages. Statistical data analyses were performed using the SPSS software (version 27.0.1) and R software (version 4.4.1). *P*-value < 0.05 was considered statistically significant.

## RESULTS

### Baseline characteristics of kidney transplant recipients

The basic characteristics of kidney transplant patients are shown in Table 1. This study included 61 kidney transplant patients, including 30 with PTDM and 31 without PTDM. In the PTDM group, 76.7% of the patients were male with an average age of 38.2 ± 11.1 years. In the control group, 77.4% of the patients were male with an average age of 37.8 ± 8.9 years. The average dialysis duration for both groups was 24 months, and >80% of the patients underwent hemodialysis. Compared to the control group, patients in the PTDM group showed a significantly higher BMI (24.9 ± 3.7 vs. 21.9 ± 3.1, *P* < 0.05). We did not observe any statistically significant differences between the two groups with regard to age, gender, dialysis type, dialysis duration, donor source, smoking, drinking, and laboratory parameters.

**TABLE 1:** Baseline characteristics of kidney transplant recipients^*a*^

Parameters	PTDM (*N* = 30)	Control (*N* = 31)	*P* value
Age, years	38.2 ± 11.1	37.8 ± 8.9	ns
Gender, Male, N (%)	23 (76.7)	24 (77.4)	ns
BMI, kg/m^2^	24.9 ± 3.7	21.9 ± 3.1	*P* < 0.05
Types of dialysis			
Hemodialysis, N (%)	24 (80.0)	27 (87.1)	ns
Peritoneal dialysis, N (%)	6 (20.0)	4 (12.9)	ns
Dialysis duration, months	24 (9.5, 36.0)	24 (9.0, 36.0)	ns
Source of Donor			
DD, N (%)	25 (83.3)	20 (64.5)	ns
LD, N (%)	5 (16.7)	11 (35.5)	ns
Hypertension, N (%)	20 (66.7)	27 (87.1)	ns
Smoking, N (%)	12 (40.0)	8 (25.8)	ns
Drinking alcohol, N (%)	7 (23.3)	5 (16.1)	ns
GFR, mL/min	56.1 ± 22.9	57.7 ± 14.7	ns
Cr, μmoI/L	98.5 (88.5, 159.7)	111 (94, 132.2)	ns
Albumin, g/L	45.7 ± 3.8	46.3 ± 3.2	ns
UA, μmol/L	336.3 ± 84.9	336.5 ± 88.9	ns
Urea, mmol/L	7.3 (5.1, 8.9)	6.5 (6.2, 7.7)	ns
Cystatin C, mg/L	1.61 (1.3, 1.8)	1.45 (1.3, 1.7)	ns
ALP, U/L	83 (68.0, 109.2)	81 (65.0, 96)	ns
TC, mmol/L	4.5 ± 1.1	4.6 ± 1.2	ns
TG, mmol/L	1.6 (1.2, 2.6)	1.5 (1.2, 1.9)	ns
HDL, mmol/L	1.3 (1.1, 1.6)	1.4 (1.1, 1.6)	ns
LDL, mmol/L	2.6 ± 0.8	2.7 ± 1.0	ns
Hb, g/L	145.2 ± 24.2	150.3 ± 23.7	ns
PLT, 10^9^/L	205.1 ± 64.9	185.6 ± 47.1	ns
WBC, 10^9^/L	7.9 ± 1.8	7.7 ± 1.7	ns
Ratio of lymphocytes, %	1.8 (1.4, 2.4)	1.7 (1.1, 2.4)	ns
Tacrolimus concentration, ng/mL	8.3 ± 3.1	7.6 ± 1.9	ns

^*a*^ BMI: body mass index; DD: donor of death; LD: living donor; GRF: glomerular filtration rate; Cr: creatinine; ALP: alkaline phosphatase; UA: uric acid; TC: total cholesterol; TG: triglycerides; HDL: high-density lipoprotein; LDL: low-density lipoprotein; Hb: hemoglobin; PLT: blood platelets; WBC: white blood cells; ns: not significant(P>0.05).

### Composition of oral microbiota in the PTDM patients

**Fig 1:**
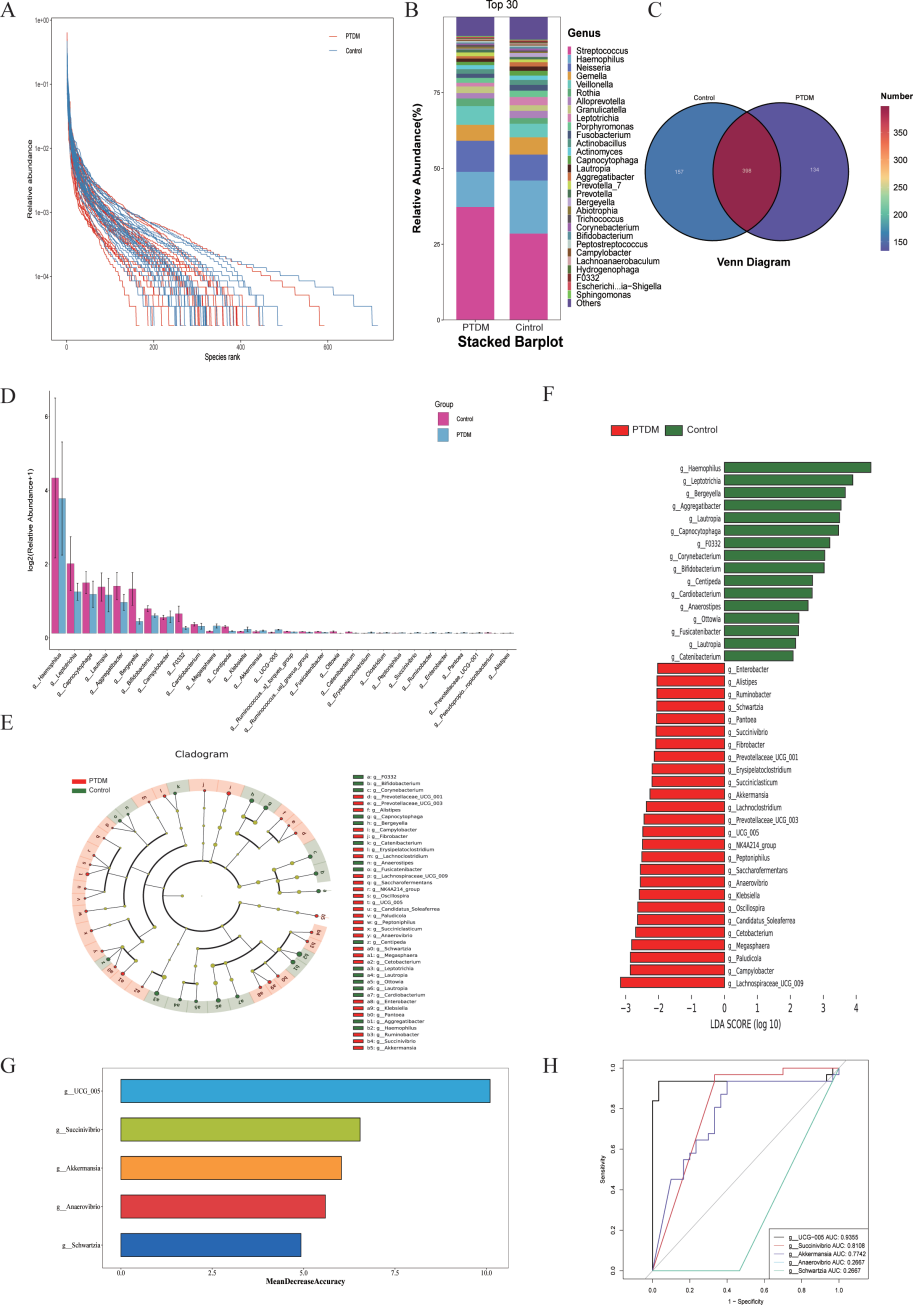
Compositional characteristics of oral microorganisms in the diabetic recipients after renal transplantation. (**A**) The species rank abundance curve depicts the species richness and species evenness in the control and PTDM groups. (**B**) Stacked bar charts demonstrate the differences in the composition and abundance of the oral microbes at the genus level in the control and PTDM groups. (**C**) Venn diagrams show the number of common and unique microbial species in the control and PTDM groups. (**D**) Histograms show the relative abundances of oral microorganisms in different subgroups. (**E**) Evolutionary branching plots show significant differences in the oral microorganisms between the PTDM and control groups. (**F**) Histograms show the distribution of the significantly distinct species between the two groups with LDA scores > 2.5 and *P* < 0.05 as threshold parameters. (**G**) Identification of the top five oral microbial traits related to PTDM using the random forest algorithm. (**H**) ROC curve analysis of the top five oral microbial species predicted by the random forest algorithm.

To determine the association between the oral microbiota and PTDM, we sequenced the V3-V4 region of the 16S rDNA gene from the oral samples of both the PTDM and the control groups. Based on the species-level abundance analysis, both groups demonstrated sufficient species richness and high abundance to support an in-depth investigation of the characteristics of the oral microbiota (Fig. 1A). There were no statistically significant differences in α-diversity and β-diversity between the PTDM group and the control group (Fig. S1A through C). At the genus level, the relative proportions of oral microbes and their contributions to the respective groups are shown in Fig. 1B. *Streptococcus*, *Hemophilus*, *Neisseria*, and *Gemella* were the most prevalent genera in both the groups (Fig. 1B). We annotated 689 species, including 134 species unique to the PTDM group, 157 species unique to the control group, and 398 species shared between the two groups (Fig. 1C).

Subsequently, we used Fisher’s exact test to identify the top 30 species with notable differences in abundance. Compared to the control group, the PTDM group showed increased relative abundances of the *Hemophilus*, *Leptotrichia*, *Capnocytophaga*, *Lautropia*, *Aggregatibacter*, *Bergeyella*, and *Bifidobacterium* genera and reduced relative abundances of the *Campylobacter*, *Megasphera*, and *Klebsiella* genera (Fig. 1D). To further confirm the specific species associated with PTDM, we performed the LEfSe analysis and obtained an LD score of 2.5. The histogram of LDA values and the cladogram of different groups (Fig. 1E and F) demonstrated that the *Campylobacter*, *Paludicola*, *Megasphera*, *Cetobacterium*, *Candidatus*_*Soleaferrea*, and *Oscillospira* genera were specific to the PTDM group.

Machine learning (random forest algorithm) is a suitable and effective approach for identifying species associated with microbial differences in studies with a limited sample size. We focused on microbes selected by combining the biomarkers selected by analysis with the random forest algorithm and the results of the LEfSe analysis. We used the random forest analysis to identify the top five microbial features (*Succinivibrio*, *Akkermansia*, *Anaerovibrio*, *Schwartzia*, and *UCG_005*) with significant differences between the groups and confirmed their diagnostic potential using the receiver operating characteristic (ROC) curve results (Fig. 1G and H). The results showed a high degree of consistency between the random forest analysis and the LEfSe analysis. The top five microbes were as follows: *genus UCG−005* (AUC = 0.9355), *Succinivibrio* (AUC = 0.8108), *Akkermansia* (AUC = 0.7742), *Anaerovibrio* (AUC = 0.2667), and *Schwartzia* (AUC = 0.2667). As shown, *genera UCG−005*, *Succinivibrio*, and *Akkermansia* exhibited superior diagnostic potential, thereby suggesting their potential in effectively differentiating between PTDM patients and healthy individuals.

### Serum metabolite analysis in patients with PTDM

**Fig 2:**
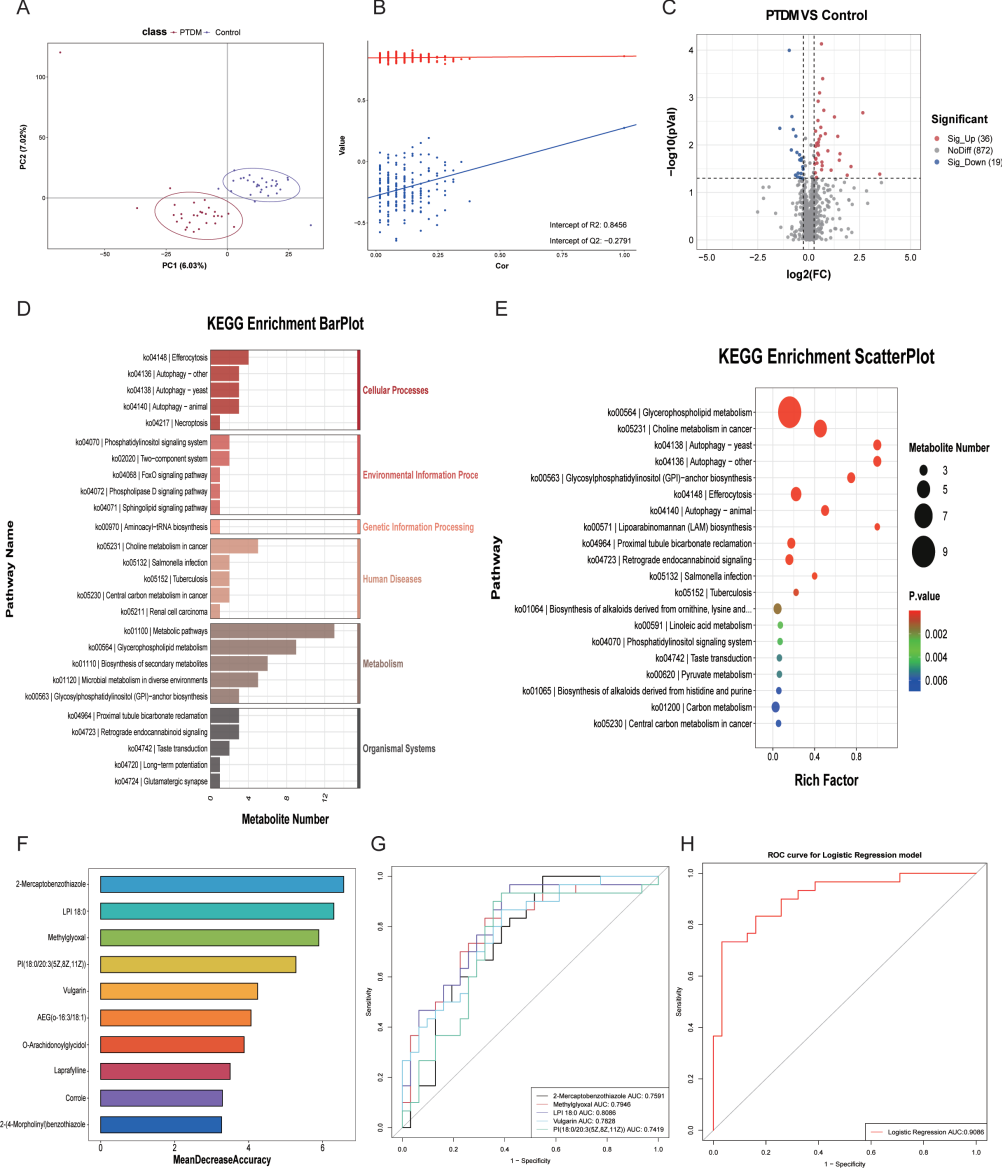
Characteristics of serum metabolites in diabetic patients after renal transplantation. (**A**) PLS-DA score plot shows the differences in the serum metabolites between the PTDM and the normal groups. (**B**) The displacement test plot shows the discriminating ability of the PLS-DA model and whether it is overfitted. (**C**) The volcano plot demonstrates the overall distribution of the differential metabolites in the PTDM group compared to the control group. (**D**) The bar graph shows the KEGG annotation and classification of the differential metabolites. (**E**) The bubble plot demonstrates the KEGG enrichment results of the differential metabolites between the two groups. (**F**) The top 10 metabolites predicted by the random forest algorithm to cause PTDM. (**G**) ROC curve analyses of the top five metabolites predicted by the random forest algorithm to cause PTDM. (**H**) ROC curve analysis using the logistic regression model for the top five metabolites and the total AUC value for the target metabolites.

**Fig 3:**
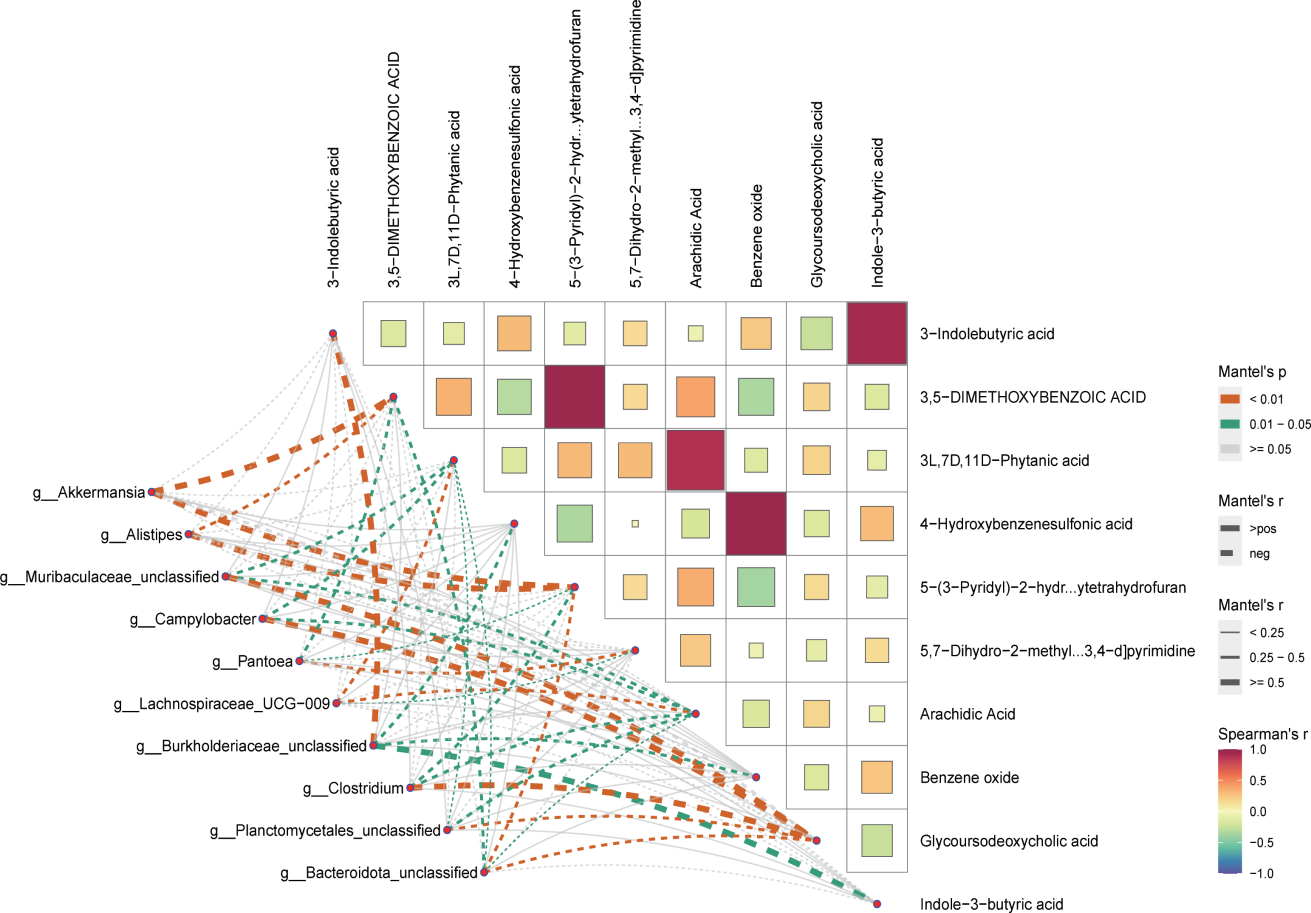
Mantel test network heatmap of the differential oral microbiota and differential metabolites. The squares in the heatmap on the right represent the strength of correlations between the metabolomes, with the red squares indicating stronger positive correlations (coefficients closer to 1) and the blue squares indicating stronger negative correlations (coefficients closer to −1). The network diagram in the lower left panel displays the correlation analysis results between the 10 differential microbial species and the 10 differential metabolites. The color represents the *P*-values. The thickness of the lines represents the correlation coefficients (r value), with a thicker line indicating a stronger correlation.

Since we observed variations in the composition of the oral microbiota between the PTDM and the control groups, we performed metabolomics of the serum samples. The orthogonal partial least squares discriminant analysis (OPLS-DA) score plot demonstrated significant differences in the serum metabolites between the normal and the PTDM groups. Permutation tests confirmed the absence of overfitting and validated the PLS-DA model (Fig. 2A and B). The LC-MS metabolomics analysis identified 927 metabolites. Among these, 36 metabolites were upregulated, and 19 metabolites were downregulated in the PTDM group compared to the control group (Fig. 3C). Subsequently, we annotated the differential metabolites using the HMDB and KEGG databases and identified 6,423 KEGG orthologs (KOs). The differential metabolites were predominantly enriched in 20 metabolic pathways, including glycerophospholipid metabolism, choline metabolism in cancer, autophagy—yeast, autophagy—other, glycosylphosphatidylinositol (GPI) anchor biosynthesis, efferocytosis, autophagy—animal, lipoarabinomannan (LAM) biosynthesis, proximal tubule bicarbonate reclamation, and retrograde endocannabinoid signaling (Fig. 2D and E).

Subsequently, we used the random forest algorithm and identified 2-mercaptobenzothiazole, lysophosphatidylcholine 18:2, methylglyoxal, PI (18:0/20:3(5Z,8Z,11Z)), Vulgarin, AEG (o-16:3/18:1), O-Arachidonoylglycidol, laprafylline, corrole, and 2-(4-Morpholinyl)benzothiazole as significant serum metabolites in the PTDM group (Fig. 2F), which included. We then performed ROC curve analysis of these serum metabolites to determine their diagnostic potential. As shown in Fig. 2G, LPI 18:0 (AUC: 0.8086), methylglyoxal (AUC: 0.7946), and Vulgarin (AUC: 0.7828) showed high diagnostic potential. Furthermore, logistic regression analysis of these five metabolites yielded a combined AUC of 0.9086 (Fig. 2H). This demonstrated that these serum metabolites effectively distinguished PTDM patients from healthy individuals and are potential diagnostic biomarkers.

### Correlation analysis between oral microbiota related to PTDM and serum metabolites

To further investigate the relationship between the oral microbiota associated with PTDM and serum metabolites, the top 10 oral microorganisms and blood metabolites with significant differences were analyzed using a Mantel test. As shown in Fig. 3, 5-(3-Pyridyl)−2-hydroxytetrahydrofuran showed significant positive correlation with the genus *Akkermansia* (r = 0.65, *P* < 0.05) and the genus *Alistipes* (r = 0.54, *P* < 0.05). Furthermore, glycoursodeoxycholic acid demonstrated significant positive correlation with the genus *Muribaculaceae_unclassified* (r = 0.56, *P* < 0.05), genus *Campylobacter* (r = 0.52, *P* < 0.05), genus *Clostridium* (r = 0.50, *P* < 0.05), genus *Planctomycetales_unclassified* (r = 0.45, *P* < 0.05), and genus *Bacteroidota_unclassified* (r = 0.44, *P* < 0.05). Moreover, 3,5-dimethoxybenzoic acid showed significant positive correlation with the genus *Akkermansia* (r = 0.57, *P* < 0.05) and genus *Alistipes* (r = 0.49, *P* < 0.05). We also observed a negative correlation of 4-Hydroxybenzenesulfonic acid with genus *Pantoea*, genus *Clostridium*, genus *Akkermansia*, and genus *Bacteroidota_unclassified*, but this correlation was not statistically significant. These results demonstrated an interactive relationship between the oral microbiota and serum metabolites in PTDM.

### Relationship between PTDM-associated oral microbiota, serum metabolites, and clinical indicators

**Fig 4:**
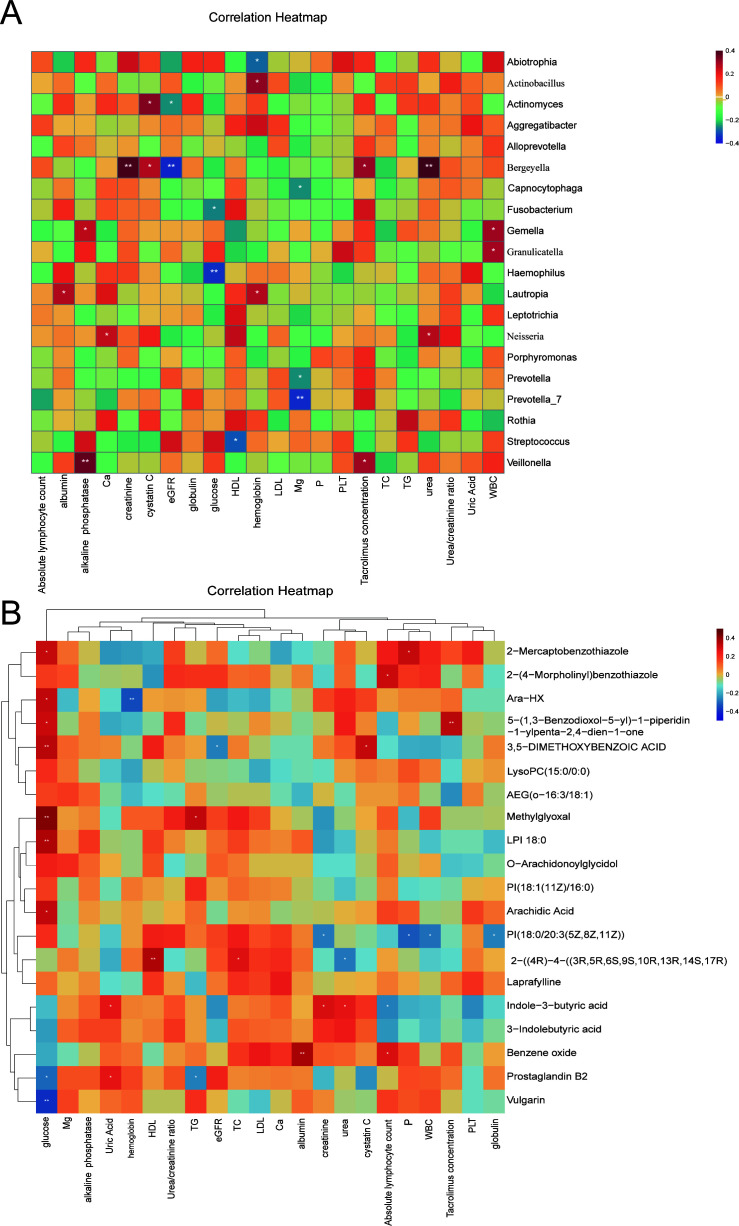
Analysis of the relationships between oral microbiota, serum metabolites, and clinical indicators in PTDM. (**A**) Relationship of the PTDM-related oral microbiota with the clinical indicators. (**B**) Relationship of the PTDM-related serum metabolites with the clinical indicators. *P ≤ 0.05, **P ≤ 0.001. The red color indicates positive correlation, and the blue color indicates negative correlation. A darker color indicates a stronger correlation. eGFR: estimated glomerular filtration rate; TG: triglyceride; TC: total cholesterol; HDL: high-density lipoprotein; LDL: low-density lipoprotein; WBC: white blood cells; PLT: platelet count.

To investigate the relationship of the PTDM-related oral microbiota and serum metabolites with the clinical indicators, we performed Spearman correlation analysis of the top 20 oral microorganisms and serum metabolites based on average abundance. Spearman correlation analysis showed significant correlation of the oral microbiota with various clinical indicators (Fig. 4A). Specifically, genus *Bergeyella* showed a positive correlation with creatinine, cystatin C, tacrolimus, and urea, and a negative correlation with eGFR. Furthermore, glucose showed a negative correlation with the *Hemophilus* and *Fusobacterium* genera. Moreover, hemoglobin levels showed a negative correlation with *Actinobacillus* and a positive correlation with *Abiotrophia*.

Serum metabolites also showed a close relationship with various clinical indicators (Fig. 4B). Indole-3-butyric acid showed positive correlation with uric acid, creatinine, and urea, and negative correlation with the absolute lymphocyte count. Furthermore, 3,5-dimethoxybenzoic acid showed a positive correlation with glucose and cystatin C and a negative correlation with eGFR. Moreover, glucose was associated with 2-mercaptobenzothiazole, Ara-HX, 5-(1,3-benzodioxol-5-yl)−1-piperidin-1-ylpenta-2,4-dien-1-one, 3,5-dimethoxybenzoic acid, and methylglyoxal.

## DISCUSSION

Recent research has highlighted the significant roles of the oral microbiota and serum metabolites in the pathophysiology of diabetes. For example, the oral microbe Actinomyces naeslundii indirectly influences the onset and progression of diabetes by affecting the levels of butyrate metabolites in the serum (35, 36). However, to date, the roles of oral microbiota and serum metabolites have not been characterized in patients with PTDM. Therefore, this study used 16S rDNA sequencing and serum metabolomics to investigate the relationship between the oral microbiota and serum metabolites in patients with PTDM.

In this study, we annotated a total of 689 oral microorganisms, including 134 species unique to the PTDM group and 157 species unique to the control group. Furthermore, metabolomics results showed upregulation of 36 serum metabolites and downregulation of 19 serum metabolites in the PTDM group compared to the control group. These changes in serum metabolites led to functional alterations in metabolic pathways such as glycerophospholipid metabolism, autophagy-yeast, and glycosylphosphatidylinositol (GPI) anchor biosynthesis. We also identified 5 oral microorganisms and 10 serum metabolites that were related to PTDM through the random forest machine learning algorithm. Among these, genera *UCG−005*, *Succinivibrio*, and *Akkermansia*, as well as serum metabolites such as LPI 18:0, methylglyoxal, and Vulgarin demonstrated significant potential as diagnostic biomarkers for PTDM. Furthermore, we observed significant correlations between the oral microbiota and serum metabolites, as well as significant associations of several clinical indicators in the PTDM patients (creatinine, cystatin C, and urea) with both the oral microbiota and the serum metabolites.

Our data demonstrated dysbiosis in the oral microbiota of the PTDM recipients. Dysbiosis in the oral microbiota has previously been reported in T2DM patients (19, 37). *Streptococcus*, *Prevotella*, and *Neisseria* are the dominant bacterial genera in the T2DM patients (37–39). This was consistent with our findings in PTDM recipients. This suggested a similarity between PTDM and T2DM regarding oral microbiota composition. Our data did not show any significant differences in the α-diversity and β-diversity of the oral microbiota between the PTDM and control groups. This was consistent with the previous findings by Wang et al. and Ana et al. (37, 38). However, the study by Saeb et al. reported a relative reduction in the biological and phylogenetic diversity of the oral microbiota in the T2DM patients (40). This was inconsistent with our results. The potential reasons for this discrepancy include a smaller sample size (*N* = 15) in the study by Saeb et al. and the essential pathophysiological differences between T2DM and PTDM (40).

Furthermore, we identified five oral microorganisms associated with PTDM through machine learning and LEfSe analysis. These were *Succinivibrio, UCG−005*, *Akkermansia*, *Anaerovibrio*, and *Schwartzia. Akkermansia* is an oval-shaped, non-spore-forming, gram-negative bacterium that is strictly anaerobic and is mainly linked to metabolic disorders, including T2DM (41, 42). *Akkermansia* alleviates diabetes by enhancing glucose metabolism and reducing inflammatory responses (43–45). *Akkermansia* improves glucose metabolism by ameliorating insulin resistance. A previous study reported a strong link between reduced levels of *Akkermansia* and increased insulin resistance (46). In the T2DM mice, administration of pasteurized Akkermansia significantly improved insulin resistance and dyslipidemia (43). Yoon et al. demonstrated that *Akkermansia*-secreted protein P9 directly interacted with intercellular adhesion molecule 2 (ICAM-2), a GLP-1 agonist, thereby inducing GLP-1 secretion and subsequently improving insulin resistance and hyperglycemia (44). Furthermore, studies have revealed that the P5 protein secreted by *Akkermansia* interacts with free fatty acid receptor 2, activating the G protein-coupled receptor (GPCR) signaling pathway. This activation subsequently enhances the synthesis and secretion of GLP-1, ultimately resulting in blood glucose reduction (47).

The altered abundance of *Akkermansia* in PTDM patients is closely associated with post-transplant immunosuppressive regimens. Calcineurin inhibitors (CNIs), a cornerstone of immunosuppression in kidney transplant recipients, are well documented to impair pancreatic β-cell insulin secretion and exacerbate insulin resistance (48, 49). Randomized controlled trials demonstrate that tacrolimus, a widely used CNI, confers a higher diabetes risk compared to cyclosporine (50). Consistent with clinical observations, tacrolimus administration reliably induces diabetes in rodent models by disrupting key β-cell signaling pathways, including CaN/NFAT and PI3K/AKT/mTOR, which collectively impair β-cell function (51–54). Notably, tacrolimus-induced diabetic rats exhibit hyperglycemia alongside significant gut microbiome remodeling, marked by reduced abundances of beneficial genera such as *Ruminococcus*, *Akkermansia*, and *Roseburia* (55). Therefore, *Akkermansia* may play a key role in ameliorating PTDM following kidney transplantation and may represent a new therapeutic target for treating PTDM in the future. Moreover, there is limited research regarding the functions of *Succinivibrio* (56), *Anaerovibrio* (57), and *Schwartzia* (58) in PTDM. Therefore, further investigations are necessary to elucidate their roles and mechanisms in PTDM.

Alterations in the composition of the oral microbiota can lead to changes in metabolism. We performed metabolomics analysis to characterize changes in the serum metabolites in the PTDM patients and found that compared to the normoglycemic group, the PTDM group showed upregulation of 36 metabolites, including AEG(o-16:3/18:1), 2-mercaptobenzothiazole, O-arachidonoylglycidol, 2-(4-morpholinyl)benzothiazole, and Laprafylline, and downregulation of 19 metabolites, including Vulgarin, 3-indolebutyric acid, prostaglandin B2, benzene oxide, and indole-3-butyric acid. Previous studies have corroborated our findings. A previous study performed non-targeted metabolomics analysis of fecal samples from 100 PTDM patients post-kidney transplantation and identified 114 significantly decreased metabolites and 8 significantly increased metabolites (59). In contrast to fecal metabolites, specific metabolites, including 2-mercaptobenzothiazole, Laprafylline, Vulgarin, prostaglandin B2, and benzene oxide, were exclusively identified in serum samples. The changes in metabolites typically lead to functional alterations. In this study, we identified 6,423 KEGG orthologs (KOs), the majority of which were related to metabolic processes, with glycerophospholipid metabolism being the most significantly enriched pathway. Previous studies have reported that upregulation of glycerophospholipid metabolism impaired the ability of insulin to inhibit hepatic gluconeogenesis (60). Zhang et al. demonstrated that overexpression of glycerol-3-phosphate acyltransferase-1 in the mouse hepatocytes reduced the binding of mTOR to its receptor. This inhibited the activity of mTOR complex 2 (mTORC2) and impaired insulin signaling (61). This supported the notion that the pathways enriched in our study were closely related to insulin resistance.

We also used machine learning algorithms and identified 10 serum metabolites that were associated with PTDM, including 2-mercaptobenzothiazole, lysophosphatidylcholine 18:2(LysoPC 18:2), methylglyoxal, PI(18:0/20:3(5Z,8Z,11Z)), Vulgarin, and AEG(o-16:3/18:1)). Recent studies have shown that 2-mercaptobenzothiazole and its derivatives inhibit α-glucosidase. Ullah et al. reported that derivatives of 2-mercaptobenzothiazole (compounds 2–4, 6–7, 9–26, 28, and 30) demonstrated significantly higher α-glucosidase inhibitory activity compared to acarbose, thereby suggesting their potential use as therapeutics for diabetes in the future (62). Consistently, large-scale population studies have identified elevated levels of LysoPC 18:2 as a significant predictor of impaired glucose tolerance and T2DM (63). Furthermore, mechanistic investigations in choline/ethanolamine phosphotransferase 1 (CEPT1)-knockout mice demonstrated that CEPT1 deficiency enhances insulin sensitivity by suppressing sarco/endoplasmic reticulum calcium ATPase-mediated calcium uptake, thereby leading to sustained activation of calcium signaling pathways (64). Vulgarin exhibits anti-diabetic effects and improves fasting blood glucose, insulin, and glycated hemoglobin levels in diabetic rats by regulating the expression of PEPCK and G-6-P genes (65). In summary, we infer that 2-mercaptobenzothiazole, LysoPC 18:2, and Vulgarin play crucial roles in improving PTDM following kidney transplantation, thereby providing new insights for future treatment strategies for PTDM.

Our study has a few limitations. First, the sample size was small. Therefore, larger multicenter studies are necessary in the future to confirm our findings. Second, this study only characterized the oral microbiota and serum metabolites associated with PTDM but did not perform in-depth animal and cell experiments to explore the specific mechanisms. Third, we analyzed the relationship of oral microbiota and metabolites with the clinical indicators but did not evaluate the underlying mechanisms. Lastly, we limited the sequencing of oral microbiota to the V3-V4 region of the 16S rRNA gene. This restricts a comprehensive understanding of the microbial community.

Future research studies should employ animal models and cellular experiments to investigate the molecular mechanisms by which oral microbiota and serum metabolites contribute to the development of PTDM. Furthermore, a deeper understanding of how the metabolites from oral microbiota influence the host metabolic pathways needs to be elucidated by integrating metabolomics, genomics, and proteomics approaches. This would unravel the pathogenesis mechanisms of PTDM, thereby providing new strategies for transplant physicians in the treatment and prevention of PTDM patients.

### Conclusions

In conclusion, oral microbiota and serum metabolites are closely related to PTDM. This study demonstrated that dysbiosis of the oral microbiota in the PTDM patients was associated with alterations in the metabolites and functional changes. Furthermore, we used the random forest algorithm to identify oral microbiota and metabolites related to PTDM. These oral microbiota and metabolites are potential diagnostic biomarkers for PTDM. These findings provide new evidence for the interplay between oral microbiota and serum metabolites. Further investigations are necessary to unravel the mechanisms underlying the development of PTDM.

## Data Availability

The 16S rRNA gene sequencing datasets generated and analyzed during this study are publicly available in the NCBI Sequence Read Archive (SRA) under the BioProject accession number PRJNA1258166. The serum metabolomics datasets generated and analyzed are publicly available in the Open Archive for Miscellaneous Data(OMIX) repository at National Genomics Data Center (NGDC) under the accession number OMIX010194.

## SUPPLEMENTAL MATERIAL

Supplemental material spectrum.00892-25-s0001.docx Tables S1 and S2; Fig. S1.

ASM does not own the copyrights to Supplemental Material that may be linked to, or accessed through, an article. The authors have granted ASM a non-exclusive, world-wide license to publish the Supplemental Material files. Please contact the corresponding author directly for reuse.
