# Supplementary material for: Integrative analyses of 16S rDNA sequencing and serum metabolomics demonstrate significant roles for the oral microbiota and serum metabolites in post-kidney transplant diabetes mellitus
Source: Microbiol Spectr. 2025 Jun 10;13(7):e00892-25. doi: 10.1128/spectrum.00892-25 (PMC12211015; doi:10.1128/spectrum.00892-25)
Supplement: Supplemental material — Tables S1 and S2; Fig. S1. [file spectrum.00892-25-s0001.docx]

**Integrative analyses of 16S rDNA sequencing and serum metabolomics demonstrates significant roles for the oral microbiota and serum metabolites in post-kidney transplant diabetes mellitus**

Chao Liu^a,b^, Sheng Chao^a^, Lei Jia^a^, Qizhen Yang^a^, Qian Chen^a^ # *, Yulin Niu^a^ # *

^a^ Department of Organ Transplantation, Affiliated Hospital of Guizhou Medical University, Guiyang 550000, Guizhou Province, China

^b^ Urinary Surgery, the Affiliated Hospital of Guizhou Medical University, Guiyang 550000, Guizhou Province, China

Address correspondence to Qian Chen, chenqian1056659393@163.com and Yulin Niu, nddnyl@126.com.

Supplementary Table and Figure

Table S1 Experimental reagents and consumables

| Reagent | manufacturers |
| --- | --- |
| Bacterial genomic DNA extraction kit | TIANGEN, China |
| Illumina library Quantification kit | Kapa Biosciences, Woburn， MA， USA |
| Pusion Hot start flex 2X Master Mix NEB M0536L | NEB M0536L |
| DL2000 DNA Maker | Takara 3427A |
| Gene colour | Beijing Jin Bo Yi GBY-1 |
| Qubit dsDNA HS Assay Kit | Invitrogen,Life technologies Q32854 |
| 50×TAE Buffer | Shanghai Sangong B548101-500 |
| AMPure XT beads | Beckman A63880 |
| Methanol | 4L/ bottle A-456-4 Fisher |
| Acetonitrile | 4L/ bottle 955-4 Fisher |
| Formic acid | 50ml/ bottle A17-50 Fisher |

Table S2 Experimental apparatus

| Apparatus | Manufacturer and Model |
| --- | --- |
| Qubit luminoscope | Invitrogen，USA |
| AMPure XT Beads | Beckman Coulter Genomics, Danvers， MA， USA |
| Agilent 2100 bioanalyzer | Agilent， USA |
| illumina NovaSeq 6000 Sequencing platform | PE250 |
| Centrifuge at room temperature | Eppendorf Centrifuge 5424 |
| Vortex oscillator | WH-861 Vortex Shaker |
| Three temperature three control constant temperature water bath | Shanghai Bosun Industrial Co., LTD DK-8D |
| PCR Amplifier | Hangzhou LANGJI SCIENTIFIC Instrument Co. LTD |
| Ultra-low temperature frozen storage box | Zhongke MEILING Low TEMPERATURE TECHNOLOGY Co. LTDDW-HL388 |
| Balance | BSA124S-CW Sartorius |
| water purification equipment | D24 UV Merck Millipore |
| chromatographic column | T3 column (100mm*2.1mm, 1.8µm) Waters |
| high resolution mass spectrometer | Q-Exactive Thermo |
| UltiMate 3000 UPLC system | Thermo Fisher Scientific, Bremen, Germany |


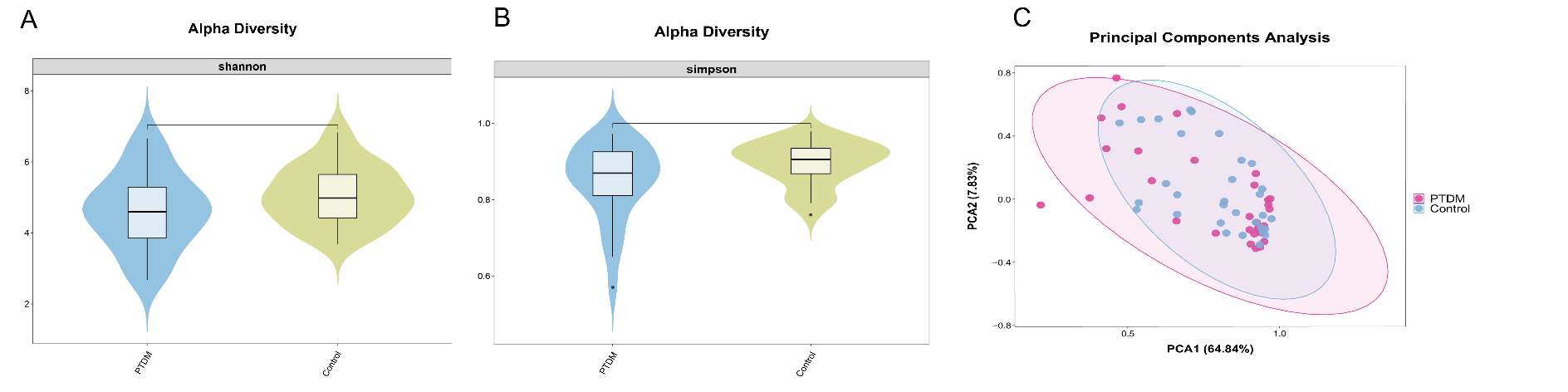


**Figure S1.** Analysis results of alpha and beta diversity for PTDM group and control group

**(A)** Violin plots of α diversity results based on the Shannon index. **(B)** Violin plots of α diversity results based on the Simpson index. **(C)** shows the β diversity results based on principal component analysis.
